# Longitudinal insights into the natural history of Type 2 diabetes among Koreans: A 20‐year community‐based prospective cohort study

**DOI:** 10.1111/joim.70010

**Published:** 2025-08-26

**Authors:** Wonsuk Choi, Joon Ho Moon, Hun Jee Choe, Howard H. Chang, Dimple Kondal, K. M. Venkat Narayan, Nam H. Cho

**Affiliations:** ^1^ Department of Internal Medicine Chonnam National University Hwasun Hospital Chonnam National University Medical School Hwasun South Korea; ^2^ Department of Biological Chemistry University of California Irvine Irvine California USA; ^3^ Department of Internal Medicine Seoul National University Bundang Hospital Seoul National University College of Medicine Seongnam South Korea; ^4^ Department of Internal Medicine Hallym University Dongtan Sacred Heart Hospital Hwaseong South Korea; ^5^ Department of Biostatistics and Bioinformatics Rollins School of Public Health, Emory University Atlanta Georgia USA; ^6^ Public Health Foundation of India New Delhi India; ^7^ Centre for Chronic Disease Control New Delhi India; ^8^ Hubert Department of Global Health Rollins School of Public Health Emory University Atlanta Georgia USA; ^9^ Emory Global Diabetes Research Center Woodruff Health Sciences Center and Emory University Atlanta Georgia USA; ^10^ Department of Preventive Medicine Ajou University School of Medicine Suwon South Korea

**Keywords:** cohort study, diabetes mellitus, impaired fasting glucose, impaired glucose tolerance, prediabetes

## Abstract

**Objective:**

To investigate the natural history of diabetes mellitus (DM) based on metabolic phenotypes of prediabetes in a community‐based prospective study.

**Methods:**

Individuals aged 40–69 years without DM were followed for 20 years. Glycemic parameters, including the 75 g oral glucose tolerance test, were assessed at baseline and biennially thereafter. Markov models were used to estimate each glycemic state's annual transition probabilities and average total length of residence.

**Results:**

Among the 7,676 participants without DM, 205 had isolated impaired fasting glucose (iIFG), and 1,753 had impaired glucose tolerance (IGT) at baseline. During the 17.5 years of follow‐up, 2,313 (30.1%) cases of DM occurred. The annual transition to DM for those with iIFG was 7.7% (95% confidence interval [CI] 6.9, 8.5) and 6.9% (95% CI 6.6, 7.3) for those with IGT. In the normoglycemia ↔ iIFG → DM model, the total length in normoglycemia was 49.4 years (95% CI 47.0, 52.1), and the length in iIFG was 6.3 years (95% CI 5.9, 6.8). In the normoglycemia ↔ IGT → DM model, the total length in normoglycemia was 34.0 years (95% CI 32.4, 35.4), and the length in IGT was 11.9 years (95% CI 11.1, 12.5).

**Conclusions:**

Individuals remained normoglycemic for long periods. However, the progression to DM occurs rapidly once prediabetes develops, regardless of the metabolic phenotype.

## Graphical abstract

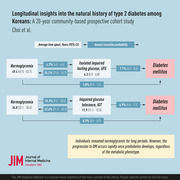

**Abbreviations**
2h‐PG: 2‐h postload glucose
ALT: alanine aminotransferase
ANOVA: analysis of variance
AST: aspartate aminotransferase
ATP: annual transition probabilities
BLSA: Baltimore Longitudinal Study of Aging
BMI: body mass index
BP: blood pressure
BUN: blood urea nitrogen
CI: confidence interval
DPP: Diabetes Prevention Program
FPG: fasting plasma glucose
GGT: gamma‐glutamyl transferase
HbA1c: glycated hemoglobin
HDL‐C: high‐density lipoprotein cholesterol
HOMA‐IR: homeostatic model assessment of insulin resistance
HOMA‐β: homeostatic model assessment of β‐cell function
IDPP: Indian Diabetes Prevention Program
IFG: impaired fasting glucose
IGT: impaired glucose tolerance
iIFG: isolated impaired fasting glucose
IR: insulin resistance
OGTT: oral glucose tolerance test
POP‐ABC: Pathobiology of Prediabetes in a Biracial Cohort
T2DM: Type 2 diabetes mellitus
TC: total cholesterol
WC: waist circumference

## Introduction

Type 2 diabetes mellitus (T2DM) is a heterogeneous disease affecting 828 million people globally [1]. Pancreatic β‐cell dysfunction and insulin resistance (IR) are key pathological mechanisms underlying the development and progression of T2DM [2]. Understanding the natural history of T2DM is essential for identifying windows of opportunity for prevention.

People go through a period of prediabetes before progressing to diabetes. Prediabetes involves two metabolic phenotypes: impaired fasting glucose (IFG) and impaired glucose tolerance (IGT). Previous studies have suggested that IFG and IGT are associated with different IR and β‐cell dysfunction patterns [3]. Individuals with IFG typically exhibit β‐cell dysfunction and hepatic IR, whereas those with IGT primarily exhibit muscle IR. Furthermore, individuals with IFG have a reduced early‐phase insulin response to oral glucose load. In contrast, individuals with IGT demonstrate defects in both early‐ and late‐phase insulin secretion. However, it remains unclear how these two distinct metabolic phenotypes of prediabetes influence the natural history of T2DM in East Asians, who exhibit unique pathophysiological characteristics, such as a higher relative contribution of β‐cell dysfunction compared to IR [4, 5]. Understanding this issue could provide valuable clinical insights into the management of prediabetes based on metabolic phenotypes in East Asia, a region where diabetes mellitus (DM) develops at a younger age and carries a higher risk of complications [6].

We investigated the natural history of T2DM based on two metabolic phenotypes of prediabetes—IFG and IGT—in community‐based prospective cohorts (*n* = 7676) of middle‐aged and older adults in Korea. The study evaluated glycemic parameters, including the 75 g oral glucose tolerance test (OGTT), biennially over a 20‐year period.

## Methods

### Study overview and participants

The Ansung–Ansan cohort study is an ongoing prospective community‐based cohort consisting of participants aged 40–69 years residing in Ansung (rural) and Ansan (urban) areas. The baseline survey was conducted in 2001–2002, with follow‐up examinations—including assessments of glycemic status such as fasting plasma glucose (FPG), glycated hemoglobin (HbA1c), and a 75 g OGTT—performed biennially. Data from 2001–2002 to 2021–2022 were analyzed in this study. More detailed information on the Ansung–Ansan cohort has been previously reported [7].

Among the 10,038 subjects, we excluded those with missing baseline demographic or laboratory measurements (*n* = 70), no follow‐up assessment of glycemic status (*n* = 13), and a history of DM (*n* = 2,028), cardiovascular disease (*n* = 178), or cancer (*n* = 73). Individuals with cardiovascular disease or cancer were excluded to minimize potential confounding, as these major medical conditions may influence glycemic homoeostasis. Finally, 7,676 subjects were included in the study (Fig. S1). Retention rates for the biennial follow‐up visits are summarized in Table S1. Missing data on glycemic status during follow‐up were addressed using the last observation carried forward approach, whereby each participant's most recently recorded glycemic state was retained until the next observation. The baseline characteristics according to study enrolment status are summarized in Table S2.

### Definition of glycemic states

Normoglycemia was defined as FPG < 100 mg/dL, 2‐h postload glucose (2h‐PG) < 140 mg/dL, and no use of anti‐DM medication. Isolated IFG (iIFG) was defined as FPG between 100 and 125 mg/dL, 2h‐PG < 140 mg/dL, and no use of anti‐DM medication. IGT was defined as FPG < 126 mg/dL, 2h‐PG between 140 and 199 mg/dL, and no use of anti‐DM medications. DM was defined as FPG ≥ 126 mg/day, 2h‐PG ≥ 200 mg/dL, HbA1c ≥ 6.5%, or the use of anti‐DM medication [8].

### Definition of cardiovascular diseases

Cardiovascular diseases were defined as coronary heart disease (including angina pectoris and myocardial infarction) and stroke. Information on cardiovascular disease was obtained from participants’ self‐reports and was validated through detailed interviews conducted during regular biennial follow‐ups.

### Baseline assessment of the covariates

Demographic data, including age, sex, education level (divided into low, lower than middle school; middle, middle school; and high, higher than middle school), household income (divided into low [<$850/month], middle [≥$850 to <$1700/month], and high [≥$1700/month]), exercise (divided into none or at least 30 min of a moderate‐to‐vigorous intensity physical activity/week), and medical history, were recorded at baseline. The smoking status was divided into never, former, and current groups. Trained nurses collected anthropometric data using standardized protocols and calibrated devices. Height, body weight, and waist circumference (WC) were measured, and body mass index (BMI) was calculated. Blood pressure (BP) was measured three times in the morning after at least 10 min of sitting. Venous blood was drawn following a fasting period of at least 8 h. For the 75 g OGTT, each participant consumed a standard dextrose solution containing 75 g of glucose dissolved in water. Additional blood samples were collected at 60 and 120 min after glucose ingestion. Immediately following collection, samples were centrifuged and stored at −80°C until analysis at a central laboratory. HbA1c levels were determined by high‐performance liquid chromatography. Plasma glucose and insulin levels were measured by the enzymatic reference method with hexokinase colorimetric assay and radioimmunoassay, respectively. Blood urea nitrogen (BUN), creatinine, albumin, aspartate aminotransferase, alanine aminotransferase (ALT), gamma‐glutamyl transferase (GGT), and lipids (total cholesterol [TC], high‐density lipoprotein cholesterol [HDL‐C], and triglycerides) were measured enzymatically. The homoeostatic model assessment was calculated for IR (HOMA‐IR) and β‐cell function (HOMA‐β) [9].

### Statistical analysis

The data are presented as mean (standard deviation) for continuous variables and count (percentage) for categorical variables. Comparisons of continuous variables between groups were conducted using analysis of variance, followed by post hoc Bonferroni corrections to adjust for multiple comparisons. Categorical variables were assessed using the chi‐square test, with post hoc pairwise comparisons conducted as necessary, also applying the Bonferroni correction. Multistate Markov models were used to calculate the annual transition probabilities (ATP) for each state, as illustrated in Figs. S2 and S3. The total estimated duration spent in each state was calculated. The outcomes of interest for each participant were iIFG, IGT, or DM. We fitted two models: (1) normoglycemia to iIFG, with the possibility of regression to normoglycemia or progression to DM (i.e., normoglycemia ↔ iIFG → DM); and (2) normoglycemia to IGT, with the possibility of regression to normoglycemia or progression to DM (normoglycemia ↔ IGT → DM). In the base‐case analysis, bidirectional transitions between states were assumed, allowing for regression from prediabetes to normoglycemia (Fig. S2). In the sensitivity analysis, unidirectional progression was examined, assuming no regression from prediabetes to normoglycemia (Fig. S3). Stratified analyses were performed by age (≤60 vs. >60 years), sex, and BMI (<23 kg/m^2^, 23 to <25 kg/m^2^, and ≥25 kg/m^2^) to estimate the ATP for each model. BMI categories were defined based on Asia–Pacific region thresholds, where 23–24.9 kg/m^2^ is classified as overweight and ≥25 kg/m^2^ as obese [10]. In the exploratory analysis, Cox regression models were used to estimate hazard ratios and 95% confidence intervals (CIs) for the incidence of cardiovascular disease. In the adjusted models, covariates included age, sex, smoking status, alcohol consumption, exercise, hypertension, dyslipidemia, and chronic kidney disease. A significance level of *p* < 0.05 was considered statistically significant for all analyses. The data were analyzed using the msm package in R software version 3.2.4.

## Results

### Baseline characteristics

Of the 7,676 participants included in the study, 3,603 (46.9%) were male, and the mean age at baseline was 51.5 years. Among the 1,753 participants in the IGT group, 201 (11.5%) had both IFG and IGT. Baseline characteristics by glycemic status are presented in Table 1. Compared to the normoglycemia group, the iIFG and IGT groups were significantly associated with higher weight, BMI, WC, systolic and diastolic BP, HbA1c, fasting glucose, HOMA‐IR, albumin, ALT, GGT, and TC levels, as well as a higher frequency of hypertension and dyslipidemia. These groups showed lower HOMA‐β and estimated glomerular filtration rate levels. When compared to the iIFG group, the IGT group was significantly associated with older age, a lower proportion of males, a higher frequency of low education, low income, and never smoking. Additionally, the IGT group had lower weight, BMI, WC, systolic and diastolic BP, fasting glucose, HOMA‐IR, albumin, and HDL‐C levels, but higher 2‐h glucose and HOMA‐β levels. The median follow‐up period was 17.5 years (interquartile range: 7.8–19.7 years), during which 2,313 (30.1%) cases of DM occurred.

**Table 1:** Baseline characteristics of participants in normoglycemia, iIFG and IGT

Characteristics	Normoglycemia (*n* = 5,718)	iIFG (*n* = 205)	IGT (*n* = 1,753)
Age, years	51.2 ± 8.7	50.4 ± 8.3	52.9 ± 8.9^b,^ ^c^
Gender (men)	2718 (47.5)	146 (71.2)^a^	739 (42.2)^b,c^
Education			
Low	1755 (31.0)	44 (21.6)^a^	618 (35.5)^b^, ^c^
Mid	3131 (55.2)	127 (62.3)	918 (52.7)
High	783 (13.8)	33 (16.2)	207 (11.9)
Income per month, USD			
Low (<850)	1897 (33.8)	47 (22.9)^a^	617 (35.7)^c^
Mid (850–1700)	1688 (30.1)	56 (27.3)	503 (29.1)
High (≥1700)	2031 (36.2)	102 (49.8)	608 (35.2)
Smoking			
Never	3354 (59.6)	74 (36.8)^a^	1109 (64.1)^b^, ^c^
Former	792 (14.1)	56 (27.9)	297 (17.2)
Current	1479 (26.3)	71 (35.3)	325 (18.8)
Current alcohol drinker	1238 (22.3)	90 (44.3)^a^	389 (22.6)
Exercise			
None	3748 (65.5)	130 (63.4)	1132 (64.6)^b^
≥Once weekly	1970 (34.5)	75 (36.6)	621 (35.4)
Height, cm	160.2 ± 8.7	163.9 ± 8.3^a^	159.1 ± 8.4^b^, ^c^
Weight, kg	62.4 ± 10.0	68.5 ± 10.3^a^	63.2 ± 10.1^b^, ^c^
BMI, kg/m^2^	24.3 ± 3.0	25.5 ± 3.4^a^	24.9 ± 3.3^b^, ^c^
Waist circumference, cm	81.7 ± 8.6	85.6 ± 8.6^a^	83.2 ± 9.0^b^, ^c^
Systolic BP, mmHg	115.3 ± 17.2	120.1 ± 17.4^a^	119.1 ± 18.4^b^
Diastolic BP, mmHg	74.2 ± 11.3	78.3 ± 11.5^a^	75.8 ± 11.6^b^, ^c^
Laboratory parameters			
HbA1c, %	5.3 ± 0.3	5.5 ± 0.4^a^	5.5 ± 0.4^b^
Fasting glucose, mg/dL	82.6 ± 7.2	105.9 ± 5.4^a^	87.8 ± 9.8^b^, ^c^
2 h glucose, mg/dL	104.8 ± 20.7	110.1 ± 21.1	161.2 ± 16.0^b^, ^c^
Insulin, mU/L	24.4 ± 19.3	27.6 ± 22.1	28.3 ± 21.6^b^
HOMA‐IR	1.5 ± 1.0	2.2 ± 1.1^a^	1.7 ± 1.0^b^, ^c^
HOMA‐β	161.0 ± 156.9	69.9 ± 32.7^a^	137.3 ± 169.0^b^, ^c^
BUN, mg/dL	14.1 ± 3.6	14.4 ± 4.0^a^	14.1 ± 3.7
eGFR, mL/min/1.73 m^2^	87.7 ± 17.4	84.7 ± 19.0^a^	86.1 ± 17.5^b^
Albumin, g/dL	4.3 ± 0.3	4.5 ± 0.4^a^	4.4 ± 0.4^b^, ^c^
AST, IU/L	27.1 ± 15.3	29.7 ± 16.6	29.7 ± 21.6^b^
ALT, IU/L	24.8 ± 19.6	28.7 ± 22.8^a^	29.1 ± 27.1^b^
GGT, IU/L	29.7 ± 46.6	49.6 ± 66.1^a^	41.4 ± 81.8^b^
Total cholesterol, mg/dL	189.4 ± 34.1	199.8 ± 34.8^a^	199.2 ± 35.5^b^
HDL‐C, mg/dL	46.6 ± 10.8	48.6 ± 12.4^a^	46.4 ± 11.1^c^
Triglycerides, mg/dL	145.1 ± 88.8	156.9 ± 121.9	169.9 ± 103.7^b^
hs‐CRP, mg/L	0.2 ± 0.4	0.2 ± 0.4	0.3 ± 0.8^b^
Comorbidities			
Hypertension	693 (12.1)	49 (23.9)^a^	372 (21.2)^b^
Dyslipidemia	1333 (23.3)	68 (33.2)^a^	616 (35.1)^b^
Chronic kidney disease	268 (4.7)	11 (5.4)	117 (6.7)^b^

*Note*: All variables are expressed as mean ± SD or *n* (%).

Abbreviations: ALT, alanine aminotransferase; AST, aspartate aminotransferase; BMI, body mass index; BP, blood pressure; BUN, blood urea nitrogen; eGFR, estimated glomerular filtration rate; GGT, gamma‐glutamyl transferase; HbA1c, glycated hemoglobin; HDL‐C, high‐density lipoprotein cholesterol; HOMA‐IR, homeostatic model assessment for insulin resistance; HOMA‐β, homeostatic model assessment of beta‐cell function; hs‐CRP, high‐sensitivity C‐reactive protein; IGT, impaired glucose tolerance; iIFG, isolated impaired fasting glucose.

^a^ Significant *p* value for normoglycemia versus iIFG.

^b^ Significant *p* value for normoglycemia versus IGT.

^c^ Significant *p* value for iIFG versus IGT.

### Markov model transition probabilities

#### Normoglycemia ↔ iIFG → diabetes mellitus pathway model

**Fig. 1:**
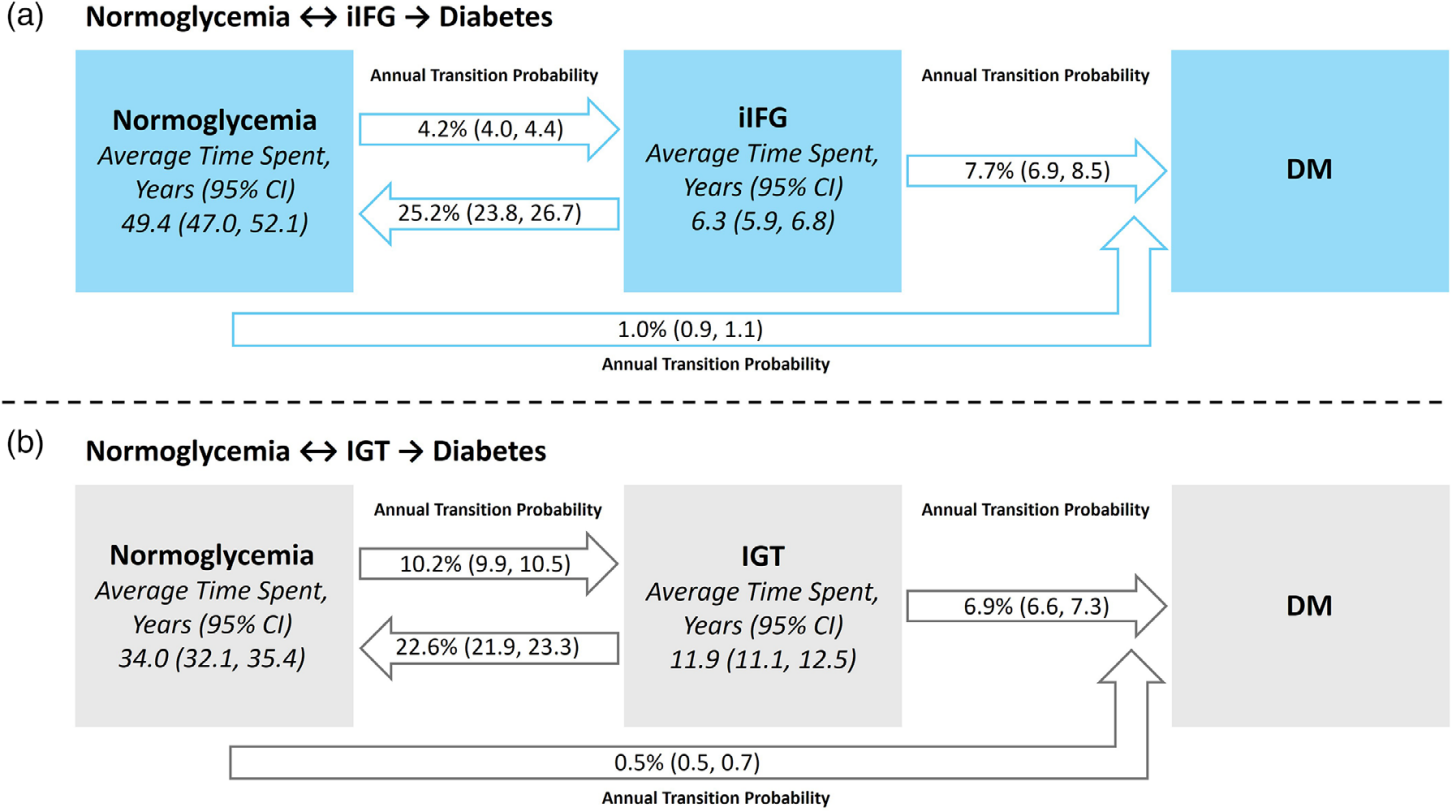
Multistate Markov models for (a) iIFG and (b) IGT. The annual probability of remaining in the same state or transitioning to the next stage (bidirectional). CI, confidence interval; DM, diabetes mellitus; iIFG, isolated impaired fasting glucose; IGT, impaired glucose tolerance.

The estimated annual probabilities of remaining in the normoglycemia and iIFG states were 94.8% (95% CI 94.6, 95.0) and 67.1% (95% CI 65.6, 68.7), respectively (Table 2). The annual probability of transitioning from normoglycemia to iIFG was 4.2% (95% CI 4.0, 4.4), whereas the probability of transitioning from iIFG to normoglycemia was 25.2% (95% CI 23.8, 26.7). The probability of transitioning from iIFG to DM was 7.7% (95% CI 6.9, 8.5). The estimated total duration spent in normoglycemia and iIFG was 49.4 years (95% CI 47.0, 52.1) and 6.3 years (95% CI 5.9, 6.8), respectively (Fig. 1a).

**Table 2:** Normoglycemia ↔ iIFG → DM multistate Markov model annual probability of transition across states (overall and stratified by age, sex, and BMI) (bidirectional)

	*n*	Annual transition probabilities, % (95% CI)
Overall		
Normoglycemia → normoglycemia	22,388	94.8 (94.6, 95.0)
Normoglycemia → iIFG	1,749	4.2 (4.0, 4.4)
Normoglycemia → DM	924	1.0 (0.9, 1.1)
iIFG → iIFG	901	67.1 (65.6, 68.7)
iIFG → normoglycemia	1,059	25.2 (23.8, 26.7)
iIFG → DM	320	7.7 (6.9, 8.5)
Age ≤60 years		
Normoglycemia → normoglycemia	18,673	94.9 (94.7, 95.1)
Normoglycemia → iIFG	1,460	4.1 (3.9, 4.4)
Normoglycemia → DM	739	1.0 (0.9, 1.1)
iIFG → iIFG	809	68.3 (66.5, 70.0)
iIFG → normoglycemia	886	24.2 (22.7, 25.8)
iIFG → DM	272	7.6 (6.8, 8.5)
Age >60 years		
Normoglycemia → normoglycemia	3,715	94.2 (93.6, 94.7)
Normoglycemia → iIFG	289	4.4 (3.9, 5.0)
Normoglycemia → DM	185	1.4 (1.1, 1.7)
iIFG → iIFG	92	59.0 (54.3, 63.8)
iIFG → normoglycemia	173	32.2 (28.0, 36.7)
iIFG → DM	48	8.8 (6.5, 11.7)
Male participants		
Normoglycemia → normoglycemia	10,008	93.2 (92.8, 93.5)
Normoglycemia → iIFG	1,110	5.7 (5.4, 6.1)
Normoglycemia → DM	447	1.1 (0.9, 1.3)
iIFG → iIFG	669	68.5 (66.6, 70.4)
iIFG → normoglycemia	687	24.8 (23.1, 26.7)
iIFG → DM	195	6.7 (5.8, 7.6)
Female participants		
Normoglycemia → normoglycemia	12,380	96.1 (95.8, 96.3)
Normoglycemia → iIFG	639	2.9 (2.7, 3.2)
Normoglycemia → DM	477	1.0 (0.9, 1.2)
iIFG → iIFG	232	63.3 (60.3, 66.3)
iIFG → normoglycemia	372	26.9 (24.3, 29.7)
iIFG → DM	125	9.8 (8.3, 11.8)
BMI < 23 kg/m^2^		
Normoglycemia → normoglycemia	8,536	95.5 (95.1, 95.8)
Normoglycemia → iIFG	553	3.7 (3.3, 4.0)
Normoglycemia → DM	263	0.9 (0.7, 1.0)
iIFG → iIFG	222	62.2 (58.8, 65.4)
iIFG → normoglycemia	345	31.1 (28.0, 34.4)
iIFG → DM	74	6.7 (5.3, 8.4)
23 kg/m^2^ ≤ BMI < 25 kg/m^2^		
Normoglycemia → normoglycemia	6,113	95.0 (94.6–95.4)
Normoglycemia → iIFG	454	4.1 (3.7–4.5)
Normoglycemia → DM	226	0.9 (0.8–1.1)
iIFG → iIFG	224	65.8 (62.4–68.9)
iIFG → normoglycemia	285	26.9 (23.9–30.0)
iIFG → DM	73	7.3 (5.9–9.1)
BMI ≥ 25 kg/m^2^		
Normoglycemia → normoglycemia	7,699	93.9 (93.5–94.2)
Normoglycemia → iIFG	740	4.8 (4.5–5.2)
Normoglycemia → DM	434	1.3 (1.1–1.5)
iIFG → iIFG	452	70.2 (68.0–72.3)
iIFG → normoglycemia	428	21.4 (19.5–23.5)
iIFG → DM	173	8.4 (7.2–9.9)

Abbreviations: BMI, body mass index; DM, diabetes mellitus; iIFG, isolated impaired fasting glucose.

#### Normoglycemia ↔ IGT → diabetes mellitus pathway model

The estimated annual probabilities of remaining in normoglycemia and IGT were 89.3% (95% CI 88.9, 89.5) and 70.5% (95% CI, 69.7, 71.2), respectively (Table 3). The probability of transitioning from normoglycemia to IGT was 10.2% (95% CI 9.9, 10.5), whereas the annual probability of transitioning from IGT to normoglycemia was 22.6% (95% CI 21.9, 23.3). The annual probability of transitioning from IGT to DM was 6.9% (95% CI 6.6, 7.3). The estimated total duration spent in normoglycemia and IGT was 34.0 years (95% CI 32.1, 35.4) and 11.9 years (11.1, 12.5), respectively (Fig. 1b).

**Table 3:** Normoglycemia ↔ IGT → DM multistate Markov model annual probability of transition across states (overall and stratified by age, sex, and BMI) (bidirectional)

	*n*	Annual transition probabilities, % (95% CI)
Overall		
Normoglycemia → normoglycemia	22,332	89.3 (88.9, 89.5)
Normoglycemia → IGT	4,716	10.2 (9.9, 10.5)
Normoglycemia → DM	704	0.5 (0.5, 0.7)
IGT → IGT	4,132	70.5 (69.7, 71.2)
IGT → normoglycemia	3,378	22.6 (21.9, 23.3)
IGT → DM	1,242	6.9 (6.6, 7.3)
Age ≤60 years		
Normoglycemia → normoglycemia	18,567	89.5 (89.1, 89.8)
Normoglycemia → IGT	3,901	10.0 (9.7, 10.4)
Normoglycemia → DM	528	0.5 (0.4, 0.7)
IGT → IGT	3,497	71.2 (70.4, 72.1)
IGT → normoglycemia	2,765	22.1 (21.3, 22.9)
IGT → DM	1,005	6.6 (6.3, 7.0)
Age >60 years		
Normoglycemia → normoglycemia	3,765	88.2 (87.3, 88.9)
Normoglycemia → IGT	815	10.9 (10.2, 11.7)
Normoglycemia → DM	176	0.9 (0.7, 1.3)
IGT → IGT	635	67.0 (64.9, 69.1)
IGT → normoglycemia	613	24.9 (23.0, 26.8)
IGT → DM	237	8.1 (7.2, 9.2)
Male participants		
Normoglycemia → normoglycemia	10,012	89.5 (89.0, 90.0)
Normoglycemia → IGT	2,079	9.9 (9.5, 10.4)
Normoglycemia → DM	358	0.6 (0.5, 0.8)
IGT → IGT	1,766	70.7 (69.5, 71.8)
IGT → normoglycemia	1,421	21.8 (20.7, 22.8)
IGT → DM	596	7.6 (7.0, 8.2)
Female participants		
Normoglycemia → normoglycemia	12,320	89.1 (88.6, 89.5)
Normoglycemia → IGT	2,637	10.4 (10.0, 10.8)
Normoglycemia → DM	346	0.5 (0.4, 0.7)
IGT → IGT	2,366	70.4 (69.3, 71.5)
IGT → normoglycemia	1,957	23.2 (22.2, 24.2)
IGT → DM	646	6.4 (6.0, 6.8)
BMI < 23 kg/m^2^		
Normoglycemia → normoglycemia	8,622	90.9 (90.4, 91.4)
Normoglycemia → IGT	1,397	8.5 (8.1, 9.0)
Normoglycemia → DM	225	0.5 (0.4, 0.7)
IGT → IGT	944	65.6 (63.8, 67.3)
IGT → normoglycemia	1,087	28.3 (26.8, 30.0)
IGT → DM	271	6.0 (5.4, 6.8)
23 kg/m^2^ ≤ BMI < 25 kg/m^2^		
Normoglycemia → normoglycemia	6,093	89.5 (88.7–90.0)
Normoglycemia → IGT	1,283	10.1 (9.5–10.6)
Normoglycemia → DM	162	0.4 (0.4–1.1)
IGT → IGT	1,156	71.6 (70.1–73.1)
IGT → normoglycemia	908	22.2 (20.8–23.7)
IGT → DM	300	6.2 (5.6–6.8)
BMI ≥ 25 kg/m^2^		
Normoglycemia → normoglycemia	7,574	87.3 (86.5–87.7)
Normoglycemia → IGT	2,025	12.1 (11.6–12.7)
Normoglycemia → DM	316	0.6 (0.6–1.2)
IGT → IGT	2,023	72.4 (71.3–73.6)
IGT → normoglycemia	1,371	19.9 (18.8–21.0)
IGT → DM	669	7.7 (7.2–8.2)

Abbreviations: BMI, body mass index; DM, diabetes mellitus; IGT, impaired glucose tolerance.

### Subgroup analysis

#### Normoglycemia ↔ iIFG → diabetes pathway model

The annual probability of transitioning from normoglycemia to iIFG was higher in male participants (5.7% [95% CI 5.4, 6.1]) compared to female participants (2.9% [95% CI 2.7, 3.2]) (Table 2). Conversely, the annual probability of transition from iIFG to DM was higher in female participants (9.8% [95% CI 8.3, 11.8]) than in male participants (6.7% [95% CI 5.8, 7.6]). Additionally, the annual probability of transitioning from normoglycemia to iIFG was higher among individuals with a BMI ≥ 25 kg/m^2^ (4.8% [95% CI 4.5, 5.2]) compared to those with a BMI < 23 kg/m^2^ (3.7% [95% CI 3.3, 4.0]).

#### Normoglycemia ↔ IGT → diabetes pathway model

The annual probability of transition from IGT to DM was higher for individuals aged >60 years (8.1% [95% CI 7.2, 9.2]) compared to those aged ≤60 years (6.6% [95% CI 6.3, 7.0]) (Table 3). Male participants had a higher annual probability of transitioning from IGT to DM (7.6% [95% CI 7.0, 8.2]) than female participants (6.4% [95% CI 6.0, 6.8]). The annual probability of transitioning from normoglycemia to IGT increased with higher BMI categories: 8.5% (95% CI, 8.1–9.0) for individuals with a BMI < 23 kg/m^2^, 10.1% (95% CI, 9.5–10.6) for those with a BMI between 23 and <25 kg/m^2^, and 12.1% (95% CI, 11.6–12.7) for those with a BMI ≥ 25 kg/m^2^. Additionally, the annual probability of transitioning from IGT to DM was higher among individuals with a BMI ≥ 25 kg/m^2^ (7.7% [95% CI, 7.2–8.2]) compared to those with a BMI < 23 kg/m^2^ (6.0% [95% CI, 5.4–6.8]).

### Sensitivity analysis (unidirectional models)

In the sensitivity analysis, the ATPs based on the unidirectional models were consistent with those observed in the base‐case bidirectional models (Tables S3 and S4 and Fig. S4).

### Exploratory analysis

In the exploratory analysis, the risk of cardiovascular disease was evaluated among individuals with normoglycemia, iIFG, and IGT in baseline (Table S5). After adjusting for covariates, there was no significant difference in cardiovascular disease risk in either the iIFG and IGT groups compared to the normoglycemia group.

## Discussion

In our cohort of middle‐aged and older adults from a community‐based study in Korea, the annual progression rate from prediabetes to DM was similar in both subtypes of prediabetes: iIFG and IGT. Individuals typically spent 34–49 years in a normoglycemic state, followed by a shorter duration of 6.3 years in iIFG or 11.9 years in IGT before developing diabetes (based on a bidirectional transition between normoglycemia and prediabetes). Prediabetes was recognized as a vulnerable stage, with up to 25% of individuals in both the iIFG and IGT groups returning to normoglycemia annually. Despite this reversibility, the progression to diabetes remained fast, with the iIFG group transitioning to diabetes at 7.7% annually, whereas the IGT group transitioned at 6.9% per year.

Several studies have reported high incidences of DM and prediabetes among Koreans [11, 12]. In the Korean Diabetes Prevention Study (KDPS), 59.2% of participants with newly diagnosed T2DM were identified solely by 2h‐PG criteria, whereas 4.5% were diagnosed based only on FPG levels [11]. Additionally, 88.8% of the newly diagnosed T2DM participants in the KDPS met the 2h‐PG diagnostic criteria. These findings highlight that impaired glucose disposal after an oral glucose load, characterized by reduced muscle insulin sensitivity and impaired late‐phase insulin secretion compared with IFG [3], is a key metabolic feature in newly diagnosed T2DM among Koreans. Our data also show that the prevalence of IGT (22.8%) was higher than that of iIFG (2.7%). Moreover, ATP from normoglycemia to IGT (10.2%) was higher than that from normoglycemia to iIFG (4.2%). These results led to a relatively shorter duration of normoglycemia and a longer duration of prediabetes in the normoglycemia ↔ IGT → DM pathway model, compared to the normoglycemia ↔ iIFG → DM pathway model. Given the high proportion of isolated IGT in the IGT group (88.5%) and the similar ATP for DM between the IGT and iIFG groups, these findings suggest that evaluating 2h‐PG using the 75 g OGTT is essential for screening for prediabetes and DM in Koreans [13]. A recent meta‐analysis involving participants from India, the United Kingdom, and Japan reported that conventional lifestyle interventions reduced the incidence of DM in individuals with IGT but not in those with iIFG [14]. Because IGT is more prevalent than iIFG in prediabetes among Koreans and because individuals with IGT spend a longer time in the prediabetes stage, active screening of high‐risk individuals using the 75 g OGTT and adoption of lifestyle interventions could help reduce the incidence of T2DM.

The prevalence of T2DM varies significantly across ethnic groups [15], suggesting that its natural history may differ according to ethnicity. Several studies have examined the natural history of T2DM across various ethnic populations [16, 17, 18, 19]. The Baltimore Longitudinal Study of Aging (BLSA) predominantly included Caucasian participants (over 95%) who had attended at least three examinations and undergone an OGTT within an 8‐year period [16]. This study found that among participants with normoglycemia at baseline, the annual progression rates to IFG and IGT were 1.7% and 6.4%, respectively. Among those with IFG or IGT at baseline, the annual progression rates to T2DM were 1.0% based on FPG criteria and 4.6% based on 2h‐PG criteria. A study involving Pima Indians, which followed participants with normoglycemia and IGT at baseline for approximately 5 years, found that the annual progression rates were 7.0% from normoglycemia to IGT and 8.0% from IGT to T2DM [17]. The Pathobiology of Prediabetes in a Biracial Cohort (POP‐ABC) Study, which included both Caucasian and African American participants who were non‐diabetic offspring of individuals with DM, found that over a 2.6‐year follow‐up period, the annual progression rate from normoglycemia to prediabetes (including IFG and IGT) was 4.2% [18]. The annual progression rates were similar between the two ethnic groups. A recent study conducted in India reported annual progression rates of 7.5% from normoglycemia to iIFG and 5.1% from normoglycemia to IGT [19]. The annual progression rates from iIFG to DM and from IGT to DM were 8.6% and 13.9%, respectively. Several previous studies, including those with intervention arms such as lifestyle modification or metformin administration, also reported the annual progression rate from prediabetes to DM. In the Diabetes Prevention Program (DPP), which included 50% Caucasians, 20% African Americans, 16% Hispanics, and less than 10% Indian Americans and Asian Americans, the annual progression rate from prediabetes (including participants with both IFG and IGT) to DM was 10.0% [20]. In the Da Qing study, which included only Chinese individuals, the annual progression rate from IGT to DM was 11.3% [21]. In the Indian Diabetes Prevention Program (IDPP), which included only Indian participants, the annual progression rate from IGT to DM was 18.3% [22]. In our study, which analyzed a community‐based cohort of middle‐aged and older adults in Korea, the annual progression rates from normoglycemia to prediabetes were 4.2% and 10.2% for the iIFG and IGT groups, respectively. The annual progression rate from normoglycemia to iIFG or IGT observed in our study is higher than that reported in the BLSA [16], the Pima Indian study [17], POP‐ABC study [18], and a recent study conducted in India [19]. However, the annual progression rate from iIFG or IGT to DM in our study is similar to Pima Indian study [17], and lower than the rate observed in the recent Indian study [19], as well as studies that included intervention arms, such as the DPP [20], the Da Qing study [21], and the IDPP [22]. The comparatively lower annual progression rate from prediabetes to DM in our study is particularly notable given that many of the aforementioned studies included intervention groups (e.g., lifestyle modifications or metformin administration), which would typically reduce annual progression rates.

Prediabetes is considered a risk factor for progression to DM and cardiovascular disease rather than a separate disease entity [8]. This represents a vulnerable state that can either revert to normoglycemia or progress to DM. Given that a transient return to normoglycemia in individuals with prediabetes is associated with a reduced risk of developing future DM, microvascular disease, and cardiovascular disease [23, 24, 25], it is crucial to understand how individuals with prediabetes transition to normoglycemia based on their metabolic phenotypes. In our study, the ATP levels from the iIFG to normoglycemia (25.2%) and from the IGT to normoglycemia (22.6%) were similar. These results differ from those of a recent study investigating the natural history of T2DM in Indians, which found a higher ATP from iIFG to normoglycemia (22.4%) compared to the ATP from IGT to normoglycemia (15.4%) [19]. The discrepant results between the two studies could be attributed to differences in the ethical background (Korean vs. Indian), participant age (mean age 51.5 vs. 38.9 years), and follow‐up duration (up to 20 vs. up to 7 years).

The strengths of our study include data from a representative sample, multiple follow‐up time points over a remarkable 20‐year period, and objective measures of glycemia obtained from biennial 75 g OGTTs. Because prediabetes is a fragile state that can transition between normoglycemia, the biennial follow‐up over 20 years in our study may precisely reflect the natural history of T2DM. Additionally, to the best of our knowledge, neither the time to progression nor the amount of time spent in each glycemic state has been calculated in any previous study on DM in Koreans. We considered bidirectional transitions from normoglycemia to iIFG or IGT in different models due to the instability of the prediabetic state. To investigate the effects of unidirectional transitions, sensitivity analyses were conducted. Regardless of whether bidirectional or unidirectional transition was assumed, the results for transition probabilities throughout states were solid. Lastly, subgroup analyses were conducted based on age, sex, and BMI.

This study has several limitations. First, because it included adults aged 40–69 years in the baseline evaluation, our findings may not be generalizable to all adults in Korea, particularly younger adults, for whom the incidence is increasing [26]. Second, because our study was based on an observational design, factors that could influence the natural history of T2DM, such as diet, exercise, and other habits, were not controlled for between participants, and their potential impact on the progression of T2DM could not be considered. Notably, individuals at risk—those who transitioned from normoglycemia to iIFG or IGT—may have been informed about their increased risk of developing DM, which could have influenced their lifestyle habits and, in turn, affected the rate of progression to DM or regression to normoglycemia. However, in our study population, more than 70% of individuals who transitioned to prediabetes maintained their prior lifestyle habits, including smoking, alcohol consumption, and physical activity. This suggests that behavioural changes were limited and less likely to have meaningfully influenced the natural history of T2DM (Table S6). Lastly, given the long follow‐up period, time‐dependent external influences and unmeasured secular changes in the environment may have affected the outcomes. However, the high retention of the cohort supports the strong internal validity of our data.

In conclusion, once prediabetes was established, both the iIFG and IGT groups progressed to DM at similar rates. Prediabetes was found as a vulnerable state, with a greater likelihood of returning to normoglycemia rather than developing into DM. Individuals at risk for DM spent an average of 34–49 years in a normoglycemic condition. Notably, the IGT group, which was more prevalent among Koreans, spent more time in the prediabetic stage than the iIFG group. These findings highlight the necessity of screening for glycemic indices, such as the 75 g OGTT, and implementing effective interventions to prevent the onset of DM.

## Author contributions

**Wonsuk Choi**: Conceptualization; data curation; formal analysis; funding acquisition; investigation; methodology; visualization; writing—original draft, writing—review and editing. **Joon Ho Moon**: Conceptualization; data curation; formal analysis; funding acquisition; investigation; methodology; writing—review and editing. **Hun Jee Choe**: Writing—review and editing. **Howard H. Chang**: Methodology; writing—review and editing. **Dimple Kondal**: Methodology; writing—review and editing. **K. M. Venkat Narayan**: Methodology; supervision; writing—review and editing. **Nam H. Cho**: Data curation; funding acquisition; resources; supervision.

## Conflict of interest statement

The authors declare no conflicts of interest.

## Funding information

The National Research Foundation (NRF) of Korea (NRF‐2022R1C1C1006021 to Wonsuk Choi and RS‐2023‐00222910 to Joon Ho Moon); the Research Program funded by the Korea Centers for Disease Control and Prevention (2001‐347‐6111‐221, 2002‐347‐6111‐221, 2003‐347‐6111‐221, 2004‐E71001‐00, 2005‐E71001‐00, 2006‐E71005‐00, 2006‐E71006‐00, 2007‐E71001‐00, 2007‐E71003‐00, 2008‐E71001‐00, 2008‐E71005‐00, 2009‐E71002‐00, 2009‐E71007‐00, 2010‐E71001‐00, 2010‐E71004‐00, 2011‐E71004‐00, 2011‐E71008‐00, 2012‐E71008‐00, 2012‐E71005‐00, 2013‐E71007‐00, 2013‐E71005‐00, 2014‐E71005‐00, 2014‐E71003‐00, 2015‐P71002‐00, 2015‐P71001‐00, 2016‐E71002‐00, 2016‐E71003‐00, 2017‐E71002‐00, 2017‐E71001‐00, 2018‐E7102‐00, 2018‐E7101‐00, 2019‐E7104‐00, 2019‐E7105‐00, 2019‐E7104‐01, 2019‐E7105‐01, 2021‐E0603‐00, 2021‐E0603‐01) to Nam H. Cho

## Ethics statement

This study was conducted in accordance with the Declaration of Helsinki, and the study protocol was approved by the ethics committee of the Korean Center for Disease Control and the Institutional Review Board of Ajou University School of Medicine (IRB number: AJIRB‐BMR‐SMP‐17‐477). All participants provided written informed consent.

## Supporting information




**Supplementary Table 1**: Retention rates for the biennial follow‐up visits.
**Supplementary Table 2**: Baseline characteristics of participants excluded from the analysis.
**Supplementary Table 3**: Normoglycemia → iIFG → DM multistate Markov model annual probability of transition across states (overall and stratified by age, sex, and BMI) (unidirectional).
**Supplementary Table 4**: Normoglycemia → IGT → DM multistate Markov model annual probability of transition across states (overall and stratified by age, sex, and BMI) (unidirectional).
**Supplementary Table 5**: Incidence rates and hazard ratios (95% CIs) for incident cardiovascular disease among individuals with normoglycemia, iIFG, and IGT at baseline.
**Supplementary Table 6**: Rates of lifestyle habit changes following transition from normoglycemia to iIFG or IGT.
**Supplementary Figure 1**: Flowchart.
**Supplementary Figure 2**: Markov model states and transition (bidirectional).
**Supplementary Figure 3**: Markov model states and transition (unidirectional).
**Supplementary Figure 4**: Multistate Markov models. The annual probability of remaining in the same state or transitioning to the next stage. (unidirectional).

## Data Availability

The data that support the findings of this study are available from the corresponding author upon reasonable request.
