# Supplementary material for: Longitudinal insights into the natural history of Type 2 diabetes among Koreans: A 20‐year community‐based prospective cohort study
Source: J Intern Med. 2025 Aug 26;298(4):336–48. doi: 10.1111/joim.70010 (PMC12459317; doi:10.1111/joim.70010)
Supplement: Supplementary file 1 — Supplementary Table 1: Retention rates for the biennial follow‐up visits. Supplementary Table 2: Baseline characteristics of participants excluded from the analysis. Supplementary Table 3: Normoglycemia → iIFG → DM multistate Markov model annual probability of transition across states (overall and stratified by age, sex, and BMI) (unidirectional). Supplementary Table 4: Normoglycemia → IGT → DM multistate Markov model annual probability of transition across states (overall and stratified by age, sex, and BMI) (unidirectional). Supplementary Table 5: Incidence rates and hazard ratios (95% CIs) for incident cardiovascular disease among individuals with normoglycemia, iIFG, and IGT at baseline. Supplementary Table 6: Rates of lifestyle habit changes following transition from normoglycemia to iIFG or IGT. Supplementary Figure 1: Flowchart. Supplementary Figure 2: Markov model states and transition (bidirectional). Supplementary Figure 3: Markov model states and transition (unidirectional). Supplementary Figure 4: Multistate Markov models. The annual probability of remaining in the same state or transitioning to the next stage. (unidirectional). [file JOIM-298-336-s001.docx]

**Supplementary Figure 1.** Flowchart


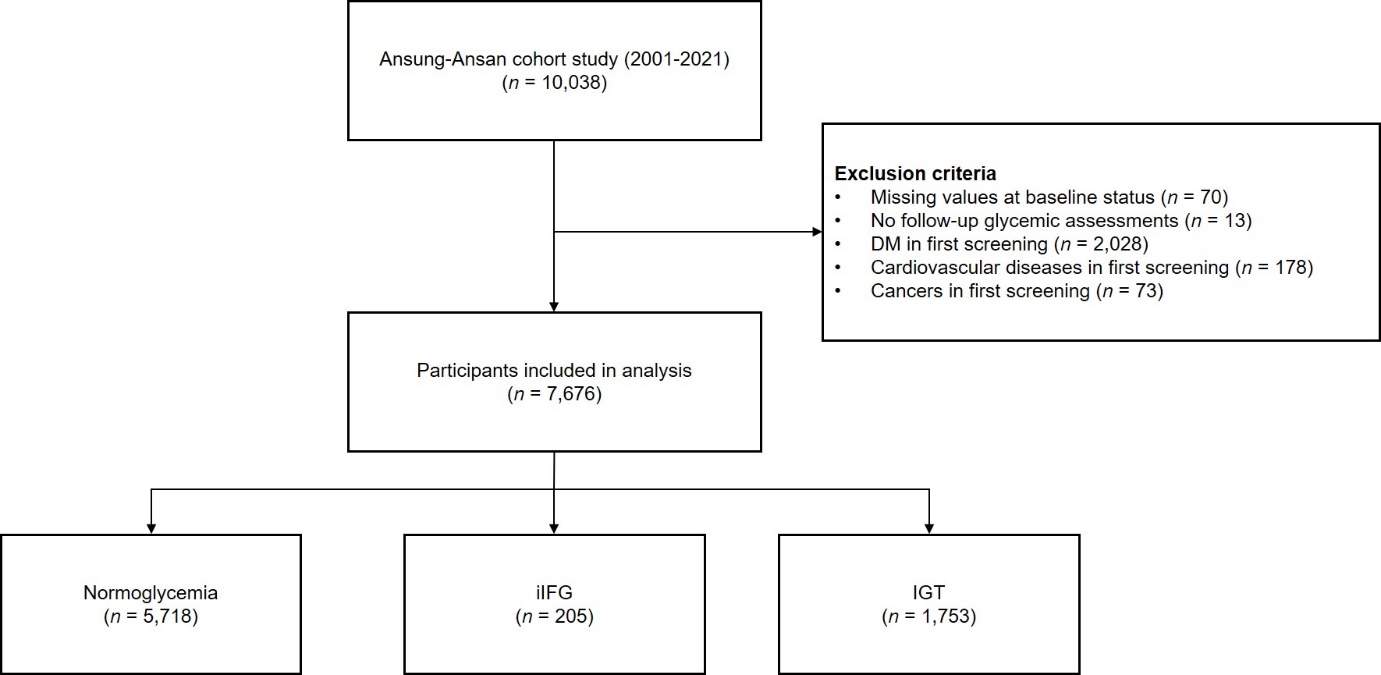


DM, diabetes mellitus; iIFG, isolated impaired fasting glucose; IGT, impaired glucose tolerance.

**Supplementary Figure 2.** Markov model states and transition (bidirectional)


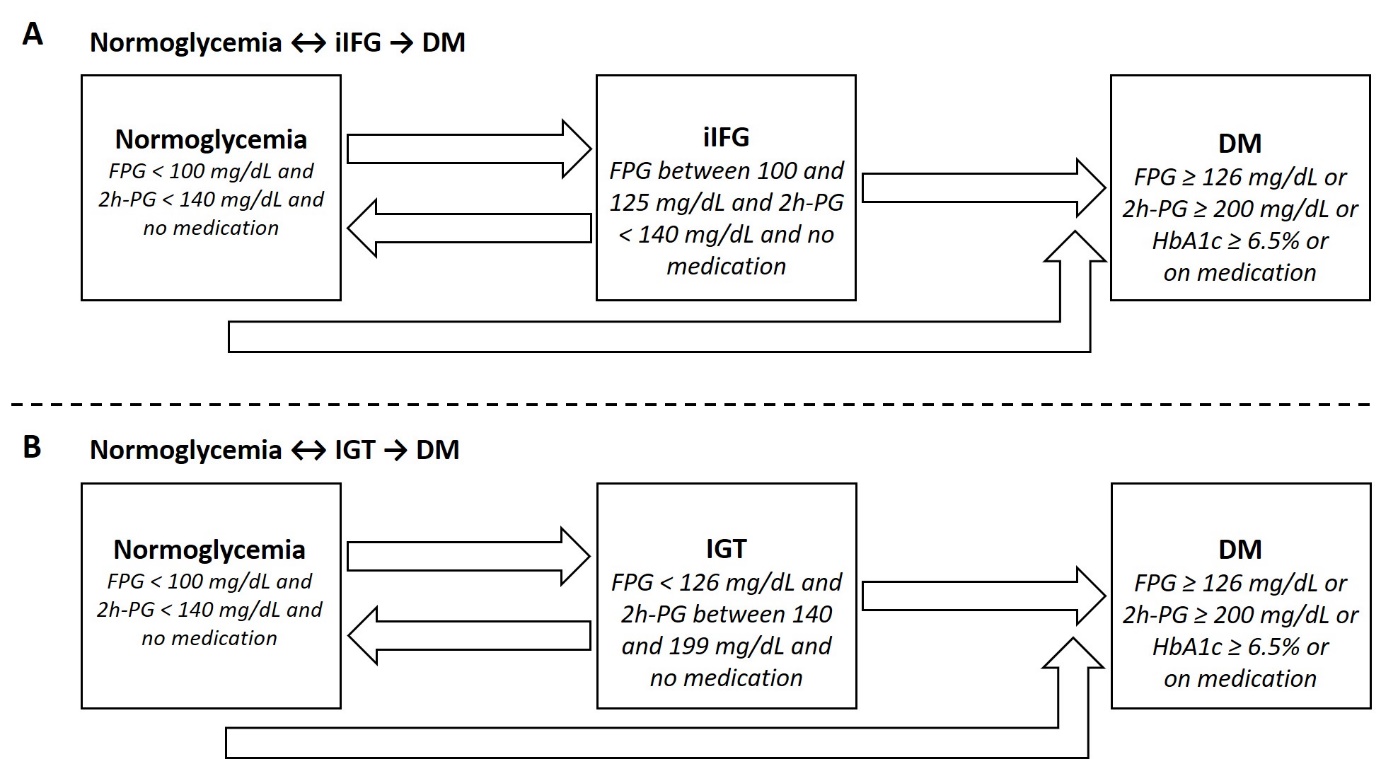


iIFG, isolated impaired fasting glucose; IGT, impaired glucose tolerance; DM, diabetes mellitus; FPG, fasting plasma glucose; 2h-PG, 2-hour postload glucose; HbA1c, glycated hemoglobin.

**Supplementary Figure 3.** Markov model states and transition (unidirectional)


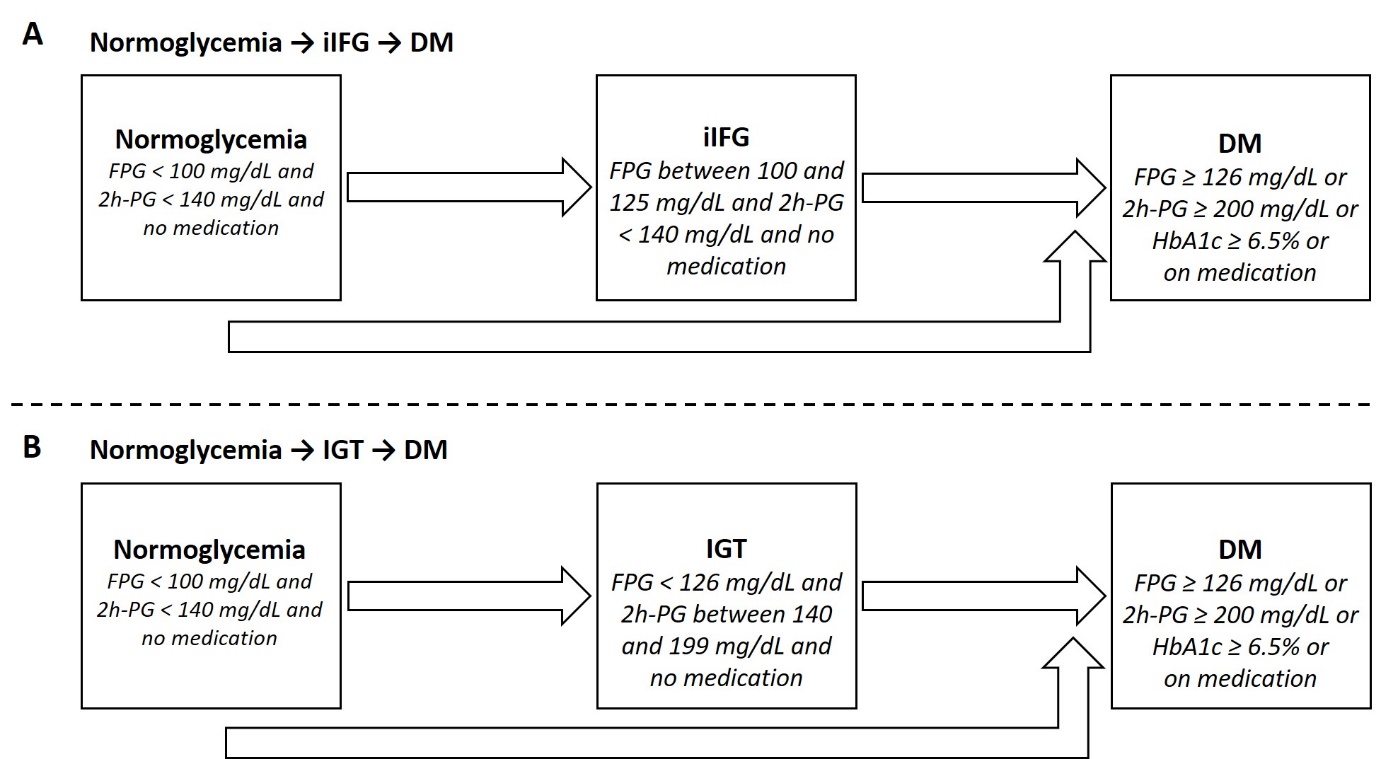


iIFG, isolated impaired fasting glucose; IGT, impaired glucose tolerance; DM, diabetes mellitus; FPG, fasting plasma glucose; 2h-PG, 2-hour postload glucose; HbA1c, glycated hemoglobin.

**Supplementary Figure 4.** Multistate Markov models. The annual probability of remaining in the same state or transitioning to the next stage. (unidirectional)


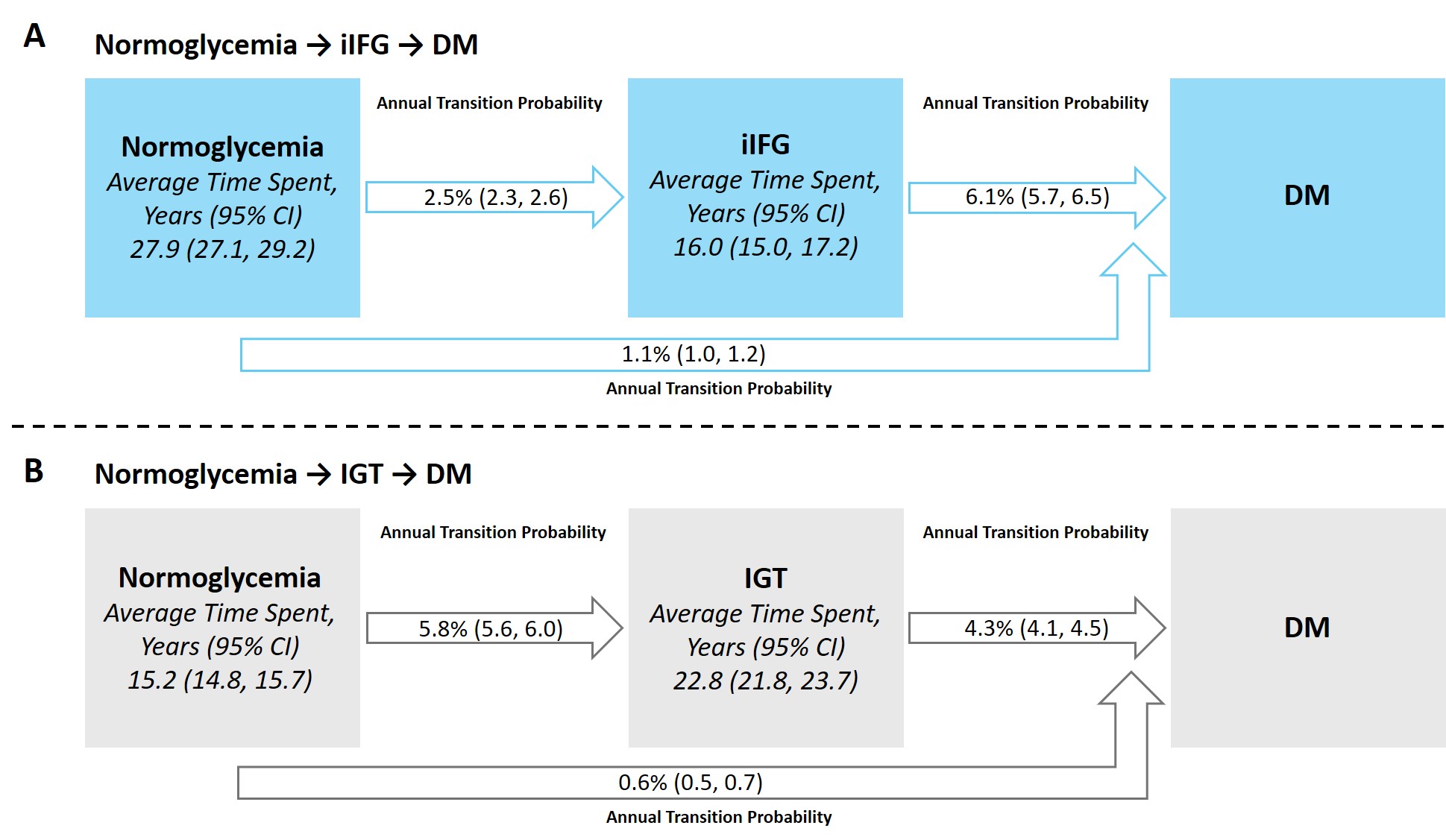


CI, confidence interval; iIFG, isolated impaired fasting glucose; IGT, impaired glucose tolerance.

| **Supplementary Table 1.** Retention rates for the biennial follow-up visits | | | | | | | | | | | |
| --- | --- | --- | --- | --- | --- | --- | --- | --- | --- | --- | --- |
| Visit | 1st visit  (Baseline) | 2nd visit | 3rd visit | 4th visit | 5th visit | 6th visit | 7th visit | 8th visit | 9th visit | 10th visit | 11th visit |
| n (%) | 7,676  (100%) | 7,203  (93.8) | 6,339  (82.6) | 5,669  (73.9) | 5,650  (73.6) | 5,357  (69.8) | 5,098  (66.4) | 5,447  (71.0) | 5,319  (69.3) | 5,089  (66.3) | 4,803  (62.6) |

| **Supplementary Table 2.** Baseline characteristics of participants excluded from the analysis | | | | |  |
| --- | --- | --- | --- | --- | --- |
| Characteristics |  |  | Subjects included in the analysis  (*n* = 7,676) | Excluded subjects  (*n* = 2,362) | |
| Age, years |  |  | 51.5 ± 8.7 | 54.7 ± 9.1 | |
| Gender (men) |  |  | 3,603 (46.9) | 1,160 (49.1) | |
| Education |  |  |  |  | |
| Low |  |  | 2,417 (31.7) | 942 (40.2) | |
| Mid |  |  | 4,176 (54.8) | 1,118 (47.7) | |
| High |  |  | 1,023 (13.4) | 284 (12.1) | |
| Income per month, USD |  |  |  |  | |
| Low (< 850) |  |  | 2,561 (33.9) | 970 (41.8) | |
| Mid (850 ~ 1700) |  |  | 2,247 (29.8) | 645 (27.8) | |
| High (≥ 1700) |  |  | 2,741 (36.3) | 703 (30.3) | |
| Smoking |  |  |  |  | |
| Never |  |  | 4,537 (60.0) | 1,298 (55.9) | |
| Former |  |  | 1,145 (15.2) | 395 (17.0) | |
| Current |  |  | 1,875 (24.8) | 628 (27.1) | |
| Current alcohol drinker |  |  | 1,717 (23.0) | 545 (23.7) | |
| Exercise |  |  |  |  | |
| None |  |  | 5,010 (65.3) | 1,489 (63.0) | |
| ≥Once weekly |  |  | 2,666 (34.7) | 873 (37.0) | |
| Height, cm |  |  | 160.1 ± 8.6 | 159.7 ± 8.7 | |
| Weight, kg |  |  | 62.7 ± 10.1 | 63.8 ± 10.2 | |
| BMI, kg/m^2^ |  |  | 24.4 ± 3.1 | 25.0 ± 3.3 | |
| Waist circumference, cm |  |  | 82.2 ± 8.7 | 84.5 ± 8.9 | |
| Systolic BP, mmHg |  |  | 116.3 ± 17.6 | 122.0 ± 19.2 | |
| Diastolic BP, mmHg |  |  | 74.7 ± 11.4 | 76.6 ± 11.6 | |
| Laboratory parameters |  |  |  |  | |
| HbA1c, % |  |  | 5.4 ± 0.4 | 6.4 ± 1.6 | |
| Fasting glucose, mg/dL |  |  | 84.4 ± 8.9 | 106.2 ± 40.0 | |
| 2h glucose, mg/dL |  |  | 117.8 ± 30.8 | 179.4 ± 83.7 | |
| Insulin, mU/L |  |  | 25.3 ± 20.0 | 24.8 ± 22.4 | |
| HOMA-IR, |  |  | 1.6 ± 1.0 | 2.1 ± 1.9 | |
| HOMA-β, |  |  | 153.1 ± 158.6 | 109.9 ± 111.4 | |
| BUN, mg/dL |  |  | 14.1 ± 3.7 | 14.6 ± 4.3 | |
| eGFR, mL/min per 1.73 m^2^ |  |  | 87.3 ± 17.5 | 85.0 ± 19.0 | |
| Albumin, g/dL |  |  | 4.3 ± 0.4 | 4.3 ± 0.4 | |
| AST, IU/L |  |  | 27.7 ± 17.1 | 30.4 ± 18.6 | |
| ALT, IU/L |  |  | 25.9 ± 21.7 | 29.7 ± 22.0 | |
| GGT, IU/L |  |  | 32.9 ± 57.4 | 47.1 ± 79.7 | |
| Total cholesterol, mg/dL |  |  | 191.9 ± 34.7 | 199.5 ± 41.0 | |
| HDL-C, mg/dL |  |  | 46.6 ± 10.9 | 45.1 ± 10.8 | |
| Triglycerides, mg/dL |  |  | 151.1 ± 94.0 | 182.9 ± 124.5 | |
| hs-CRP, mg/L |  |  | 0.2 ± 0.5 | 0.3 ± 0.6 | |
| Comorbidities |  |  |  |  | |
| Hypertension |  |  | 1,114 (14.5) | 664 (28.1) | |
| Chronic kidney disease |  |  | 396 (5.2) | 225 (9.5) | |
| Dyslipidemia |  |  | 2,017 (26.3) | 900 (38.2) | |

All variables are expressed as mean ± SD or n (%). iIFG, isolated impaired fasting glucose; IGT, impaired glucose tolerance; BMI, body mass index; BP, blood pressure; HbA1c, glycated hemoglobin; HOMA-IR, homeostatic model assessment for insulin resistance; HOMA-β, homeostatic model assessment of beta-cell function; BUN, blood urea nitrogen; eGFR, estimated glomerular filtration rate; AST, aspartate aminotransferase; ALT, alanine aminotransferase; GGT, gamma-glutamyl transferase; HDL-C, high-density lipoprotein cholesterol.

| **Supplementary Table 3.** Normoglycemia → iIFG → DM multistate Markov model annual probability of transition across states (overall and stratified by age, sex, and BMI) (unidirectional) | | | |
| --- | --- | --- | --- |
|  |  | *n* | Annual transition probabilities, % (95% CI) |
| Overall |  |  |  |
| Normoglycemia → normoglycemia |  | 20,986 | 96.5 (96.3, 96.6) |
| Normoglycemia → iIFG |  | 1,318 | 2.4 (2.3, 2.5) |
| Normoglycemia → DM |  | 837 | 1.1 (1.0, 1.2) |
| iIFG → iIFG |  | 4,247 | 93.9 (93.5, 94.3) |
| iIFG → DM |  | 734 | 6.1 (5.7, 6.5) |
| Age ≤ 60 years |  |  |  |
| Normoglycemia → normoglycemia |  | 17,497 | 96.6 (96.4, 96.7) |
| Normoglycemia → iIFG |  | 1,087 | 2.3 (2.2, 2.5) |
| Normoglycemia → DM |  | 675 | 1.1 (1.0, 1.2) |
| iIFG → iIFG |  | 3,244 | 95.8 (95.3, 96.2) |
| iIFG → DM |  | 336 | 4.2 (3.8, 4.7) |
| Age > 60 years |  |  |  |
| Normoglycemia → normoglycemia |  | 3,501 | 95.9 (95.5, 96.3) |
| Normoglycemia → iIFG |  | 238 | 2.7 (2.3, 3.0) |
| Normoglycemia → DM |  | 167 | 1.4 (1.2, 1.7) |
| iIFG → iIFG |  | 530 | 94.9 (93.6, 95.9) |
| iIFG → DM |  | 66 | 5.1 (4.1, 6.4) |
| Male participants |  |  |  |
| Normoglycemia → normoglycemia |  | 9,162 | 95.5 (95.3, 95.8) |
| Normoglycemia → iIFG |  | 810 | 3.2 (3.0, 3.5) |
| Normoglycemia → DM |  | 402 | 1.2 (1.1, 1.4) |
| iIFG → iIFG |  | 2,502 | 96.1 (95.6, 96.5) |
| iIFG → DM |  | 240 | 3.9 (3.5, 4.4) |
| Female participants |  |  |  |
| Normoglycemia → normoglycemia |  | 11,836 | 97.2 (97.0, 97.4) |
| Normoglycemia → iIFG |  | 515 | 1.7 (1.6, 1.9) |
| Normoglycemia → DM |  | 440 | 1.1 (1.0, 1.2) |
| iIFG → iIFG |  | 1,272 | 94.9 (94.1, 95.5) |
| iIFG → DM |  | 162 | 5.1 (4.5, 5.9) |
| BMI < 23 kg/m^2^ |  |  |  |
| Normoglycemia → normoglycemia |  | 8,045 | 97.1 (96.8, 97.3) |
| Normoglycemia → iIFG |  | 417 | 2.0 (1.8, 2.2) |
| Normoglycemia → DM |  | 239 | 0.9 (0.8, 1.1) |
| iIFG → iIFG |  | 1,194 | 96.5 (95.8, 97.1) |
| iIFG → DM |  | 98 | 3.5 (2.9, 4.2) |
| 23 kg/ m2 ≤ BMI < 25 kg/m2 |  |  |  |
| Normoglycemia → normoglycemia |  | 5,725 | 96.7 (96.4-96.9) |
| Normoglycemia → iIFG |  | 341 | 2.2 (2.0-2.5) |
| Normoglycemia → DM |  | 208 | 1.1 (0.9-1.3) |
| iIFG → iIFG |  | 1,010 | 96.2 (95.2-96.9) |
| iIFG → DM |  | 91 | 3.8 (3.1-4.6) |
| BMI ≥ 25 kg/m2 |  |  |  |
| Normoglycemia → normoglycemia |  | 7,188 | 95.7 (95.4-96.0) |
| Normoglycemia → iIFG |  | 566 | 2.9 (2.7-3.1) |
| Normoglycemia → DM |  | 394 | 1.4 (1.3-1.6) |
| iIFG → iIFG |  | 1,565 | 94.7 (93.9-95.3) |
| iIFG → DM |  | 213 | 5.3 (4.7-6.0) |

BMI, body mass index; iIFG, isolated impaired fasting glucose; DM, diabetes mellitus.

| **Supplementary Table 4.** Normoglycemia → IGT → DM multistate Markov model annual probability of transition across states (overall and stratified by age, sex, and BMI) (unidirectional) | | | |
| --- | --- | --- | --- |
|  |  | *n* | Annual transition probabilities, % (95% CI) |
| Overall |  |  |  |
| Normoglycemia → normoglycemia |  | 18,433 | 93.7 (93.4, 93.9) |
| Normoglycemia → IGT |  | 2,875 | 5.8 (5.6, 6.0) |
| Normoglycemia → DM |  | 484 | 0.6 (0.5, 0.7) |
| IGT → IGT |  | 13,250 | 95.7 (95.5, 95.9) |
| IGT → DM |  | 1,462 | 4.3 (4.1, 4.5) |
| Age ≤ 60 years |  |  |  |
| Normoglycemia → normoglycemia |  | 15,251 | 93.8 (93.6, 94.0) |
| Normoglycemia → IGT |  | 2,359 | 5.7 (5.5, 5.9) |
| Normoglycemia → DM |  | 367 | 0.5 (0.4, 0.6) |
| IGT → IGT |  | 11,120 | 95.9 (95.6, 96.1) |
| IGT → DM |  | 1,166 | 4.1 (3.9, 4.4) |
| Age > 60 years |  |  |  |
| Normoglycemia → normoglycemia |  | 3,182 | 92.9 (92.3, 93.4) |
| Normoglycemia → IGT |  | 516 | 6.2 (5.7, 6.8) |
| Normoglycemia → DM |  | 117 | 0.9 (0.7, 1.1) |
| IGT → IGT |  | 2,130 | 94.8 (94.2, 95.3) |
| IGT → DM |  | 296 | 5.2 (4.7, 5.8) |
| Male participants |  |  |  |
| Normoglycemia → normoglycemia |  | 8,340 | 93.6 (93.2, 93.9) |
| Normoglycemia → IGT |  | 1,323 | 5.7 (5.5, 6.1) |
| Normoglycemia → DM |  | 263 | 0.7 (0.6, 0.9) |
| IGT → IGT |  | 5,615 | 95.3 (95.0, 95.6) |
| IGT → DM |  | 691 | 4.7 (4.4, 5.0) |
| Female participants |  |  |  |
| Normoglycemia → normoglycemia |  | 10,093 | 93.7 (93.4, 94.0) |
| Normoglycemia → IGT |  | 1,552 | 5.8 (5.6, 6.1) |
| Normoglycemia → DM |  | 221 | 0.4 (0.4, 0.6) |
| IGT → IGT |  | 7,635 | 96.0 (95.7, 96.3) |
| IGT → DM |  | 771 | 4.0 (3.7, 4.3) |
| BMI < 23 kg/m^2^ |  |  |  |
| Normoglycemia → normoglycemia |  | 7,209 | 94.9 (94.6, 95.2) |
| Normoglycemia → IGT |  | 851 | 4.5 (4.2, 4.8) |
| Normoglycemia → DM |  | 158 | 0.6 (0.5, 0.7) |
| IGT → IGT |  | 3,990 | 96.6 (96.3, 96.9) |
| IGT → DM |  | 338 | 3.4 (3.1, 3.7) |
| 23 kg/ m2 ≤ BMI < 25 kg/m2 |  |  |  |
| Normoglycemia → normoglycemia |  | 4,978 | 93.7 (93.3-94.1) |
| Normoglycemia → iIFG |  | 771 | 5.8 (5.4-6.1) |
| Normoglycemia → DM |  | 116 | 0.5 (0.4-0.7) |
| iIFG → iIFG |  | 3,691 | 96.2 (95.8-96.6) |
| iIFG → DM |  | 346 | 3.8 (3.4-4.2) |
| BMI ≥ 25 kg/m2 |  |  |  |
| Normoglycemia → normoglycemia |  | 6,215 | 92.3 (91.9-92.6) |
| Normoglycemia → iIFG |  | 1,249 | 7.1 (6.8-7.5) |
| Normoglycemia → DM |  | 209 | 0.6 (0.5-0.8) |
| iIFG → iIFG |  | 5,529 | 94.7 (94.4-95.1) |
| iIFG → DM |  | 776 | 5.3 (4.9-5.6) |

BMI, body mass index; IGT, impaired glucose tolerance; DM, diabetes mellitus.

| **Supplementary Table 5.** Incidence rates and hazard ratios (95% CIs) for incident cardiovascular disease among individuals with normoglycemia, iIFG, and IGT at baseline | | | | | |
| --- | --- | --- | --- | --- | --- |
|  |  | Number of events/participants | Incidence rate per 1000 person-years (95% CI) | Unadjusted HR (95% CI) | Adjusted HR (95% CI)^a^ |
| Normoglycemia |  | 689 / 5,718 | 88 (81.5-94.8) | Ref | Ref |
| iIFG |  | 31 / 205 | 92.1 (62.6-130.7) | 1.30 (0.91- 1.86) | 1.25 (0.87- 1.80) |
| IGT |  | 248 / 1,753 | 92.1 (81-104.3) | 1.24 (1.07- 1.43) | 1.03 (0.89- 1.20) |

CI, confidence interval; HR, hazard ratio; iIFG, impaired fasting glucose; IGT, impaired glucose tolerance. ^a^Adjusted for age, sex, smoking, alcohol drinking, exercise, hypertension, dyslipidemia, and chronic kidney disease.

| **Supplementary Table 6**. Rates of lifestyle habit changes following transition from normoglycemia to iIFG or IGT | | |
| --- | --- | --- |
|  | Normoglycemia → iIFG^a^ | Normoglycemia → IGT^a^ |
| Smoking | n (%) | n (%) |
| Never -> Never | 67 (41.1) | 899 (64.3) |
| Never -> Former | 3 (1.8) | 18 (1.3) |
| Never -> Current | 0 (0.0) | 3 (0.2) |
| Former -> Former | 49 (30.1) | 255 (18.3) |
| Former -> Current | 2 (1.2) | 12 (0.9) |
| Current -> Former | 6 (3.7) | 36 (2.6) |
| Current -> Current | 36 (22.1) | 174 (12.5) |
| Alcohol |  |  |
| Non-current -> Non-current | 11 (5.4) | 172 (10.1) |
| Non-current -> Current | 39 (19.3) | 616 (36.3) |
| Current -> Non-current | 0 (0.0) | 6 (0.4) |
| Current -> Current | 152 (75.2) | 905 (53.2) |
| Exercise |  |  |
| None -> None | 6 (3.0) | 73 (4.2) |
| None -> ≥Once weekly | 59 (29.1) | 439 (25.4) |
| ≥Once weekly -> None | 1 (0.5) | 14 (0.8) |
| ≥Once weekly -> ≥Once weekly | 137 (67.5) | 1200 (69.5) |

iIFG, impaired fasting glucose; IGT, impaired glucose tolerance. ^a^This analysis was restricted to study participants who had corresponding lifestyle habit records at the visit following their transition from normoglycemia to prediabetes—that is, the visit after their initial diagnosis of prediabetes.
